# Supplementary material for: COVID-19 Vaccination and Neurological Manifestations: A Review of Case Reports and Case Series
Source: Brain Sci. 2022 Mar 18;12(3):407. doi: 10.3390/brainsci12030407 (PMC8946610; doi:10.3390/brainsci12030407)
Supplement: Supplementary file 1 [file brainsci-12-00407-s001.zip › brainsci-1634889-supplementary.pdf]

brain sciences

MDPI

Table S1. Studies with data from case reports and case series on COVID-19 vaccine and CNS associated manifestations.

Author/ country	Patient Age/Gender	Type of COVID-19 vaccine	Comorbidities	Time Duration/ Doses to neurological symptom onset	Neurological presentations	Diagnostic Modality MRI, CT, MRA/MRV, EEG, CSF analysis	Diagnosis	Management	Outcome
Malhotra H.S. et. al./ India	36 yr/ M	Astra-Zeneca	None	8 days/ first dose	Abnormal sensations in both lower limbs. Impaired vibration sense till manubrium sterni	MRI brain: Normal MRI spine: ovoid T2-hyperintense lesion that showed mild to moderate peripheral enhancement on T1-gadolinium contrast administration	Transverse myelitis	Methylprednisolone	Improved
Vegezzi E. et. al./ Italy	44 yr/ F	Astra-Zeneca	None	4 days/ first dose	Bilateral plantar feet ascending paresthesia, reduced sensation in her lower back and during micturition, reduced light touch and pinprick up to ankles and brisk deep tendon reflexes in the lower limbs	MRI Brain: Normal MRI spine with contrast: two single-metamer lesions, in the posterior paramedian cord at D7-D8 level and in the left lateral cord at D10-D11 level, the first one with mild and patchy contrast enhancement	Transverse myelitis	Methylprednisolone	Improved
Hsiao Y.T. et. al./ Taiwan	41 yr/ M	Astra-Zeneca	Type 2 DM	14 days/ first dose	Tingling sensation over T4 dermatome was experienced, followed by progressive paresthesia below T4, along with lower-limb weakness and clumsiness. Decreased lower-limb muscle power, which was more	MRI Brain: Normal MRI Spine: intramedullary-enhancing lesion over the spinal cord at the T1 to T6 vertebral levels	Transverse myelitis (TM)	Methylprednisolone	Improved

					severe over the left side				
Khan E. et. al./ India	67 yr/F	Moderna	CAD, CKD, neuropathy and previous colon rupture with colostomy	1 day/ first dose	Bilateral upper and lower extremity weakness (right more than left), paresthesia	CT Head: Normal MRI Brain: Scattered patchy foci nonspecific signal changes; MRI cervical spine: Hyperintense lesions in the upper cervical spine and cord edema extending from C1-C3 with patchy post-contrast enhancement	Transverse myelitis (TM)	Methylprednisolone, PLEX	Improved
Fujikawa P. et. al./ USA	46 yr/ F	Moderna	Vitamin B12 deficiency	2 days/ first dose	Paresthesia distal to the T10 dermatome and bilateral upper- and lower-extremity weakness, partial urinary retention	MRI Brain: Normal MRI Spine: T2-weighted MRI of the cervical spine revealed increased, nonexpansile intramedullary signal involving the central gray matter at C6-T2 without enhancement	Neuromyelitis optica Spectrum Disorder (NMOSD)	Methylprednisolone	Improved
Chen S. et. al./ China	NA/ F	Sinovac	NA	3 days/ first dose	Dizziness, unsteady walking, nausea, vomiting, weakened pharyngeal reflex, limb muscle strength	MRI Brain: Area postrema and bilateral hypothalamus lesions without gadolinium enhancement	Neuromyelitis optica Spectrum Disorder (NMOSD)	Methylprednisolone	Improved
Notghi A.A. et. al./ UK	58 yr/ M	Astra-Zeneca	Type 2 DM, Pulmonary sarcoidosis	10 days/ first dose	Progressive numbness in his lower limbs over 3 days, allodynia up to chest level, genital dysesthesia and an episode of urinary incontinence	MRI Brain: Normal MRI Spine: Myelopathic features at cervical level, showed abnormal cord signal from T2 to T10. With gadolinium contrast revealed two separate foci of enhancing myelitis, at the T3/4-disc level at the T9/10-disc level	Longitudinally Extensive Transverse Myelitis (LETM)	Methylprednisolone, PLEX	Improved

Vogrig A. et. al./ Italy	56 yr/ F	Pfizer	Post-infectious rhombencephalitis	14 days/ first dose	Unsteadiness of gait, predominantly on the left side, followed by clumsiness of the left arm. Mild weakness on left upper limb	MRI Brain: area of hyperintensity involving the left cerebellar peduncle, with modest mass effect on the fourth ventricle, no contrast enhancement	Acute Disseminated Encephalomyelitis (ADEM)	Prednisone	Improved
Etemadifar M. et. al./ Iran	34 yr/ F	Sputnik	MS	3 days/ first dose	Right hemiplegia, ataxia. She had severe imbalance and an ataxic gait	MRI Brain: Several new periventricular, juxtacortical, and brainstem, without gadolinium enhancement on T1-weighted sequences.	Relapsing Remitting Multiple Sclerosis (RRMS)	Methylprednisolone	Improved
Syed K. et. al./ USA	45 yr/ M	Moderna	Type 2 DM	8 days/ second dose	Headache with a tonic-clonic seizure	CT head: left frontal lobe intracerebral hemorrhage (ICH) along with subarachnoid hemorrhage (SAH). CT angiography: absence of opacification in the anterior 1/2 of the superior sagittal sinus, consistent with acute venous sinus thrombosis	Cerebral Venous Sinus Thrombosis with ICH, SAH	Esmolol, Nimodipine, Levetiracetam, Heparin, Coumadin	Improved
Wolf M.E. et. al./ Germany Pt. 1	22 yr/ F	AstraZeneca	BMI=26	4 days/ first dose	Frontally accentuated headaches and generalized epileptic seizures.	MRI Brain: Blood in the subarachnoid space adjacent to the falx cerebri on both sides. The superior sagittal sinus, the left-hand transverse sinus, and the sigmoid sinus were thrombosed	Cerebral venous sinus thrombosis	Levetiracetam, Enoxaparin, Dabigatran	Improved
Wolf M.E. et. al./ Germany Pt. 2	46 yr/ F	AstraZeneca	None	13 days/ first dose	Mild aphasia and hemianopia to the right	MRI Brain: Thrombotic occlusion of the superior sagittal sinus and the left-hand transverse sinus and sigmoid sinus. An acute	Cerebral Venous Sinus Thrombosis with ICH	Danaparoid, Dabigatran	Improved

						intracerebral hematoma in the left occipital lobe			
Wolf M.E. et. al./ Germany Pt. 3	36 yr/ F	AstraZeneca	None	17 days/ first dose	Severe headache, acute somnolence and right-hand hemiparesis	MRI Brain: Thrombotic occlusion of the straight sinus and a non-occlusive thrombus in the superior sagittal sinus. Congestive edema of both thalami was more pronounced on the left side	Cerebral venous sinus thrombosis	Danaparoid, Enoxaparin, Dabigatran	Improved
Schultz N.H. et. al./ Norway Pt. 1	37 yr/ F	AstraZeneca	Pollen allergy, OCP use	8 days/ NA	Headaches, visual disturbances, fever	CT head: thrombosis in the left transverse and sigmoid sinuses. Massive cerebellar hemorrhage and edema in the posterior fossa	Cerebral Venous Sinus Thrombosis with Cerebellar hemorrhage	Dalteparin, Platelet transfusion, decompressive craniectomy	Not improved, fatal
Schultz N.H. et. al./ Norway Pt. 2	42 yr/ F	AstraZeneca	Pollen allergy	10 days/ NA	Headaches, drowsiness	CT venography: Venous thrombosis with occlusion of the transverse and sigmoid sinuses and hemorrhagic infarction in the left hemisphere	Cerebral venous sinus thrombosis	Dalteparin, Methylprednisolone, IVIG, hemicraniectomy, platelet transfusion	Not improved, fatal
Schultz N.H. et. al./ Norway Pt. 3	39 yr/ F	AstraZeneca	None	10 days/ NA	Headache, abdominal pain	CT head with venography: massive thrombosis in the deep and superficial cerebral veins and right cerebellar hemorrhagic infarction	Cerebral venous sinus thrombosis	Dalteparin, Prednisolone, IVIG	Improved
Schultz N.H. et. al./ Norway Pt. 4	54 yr/ F	AstraZeneca	HTN, HRT use	7 days/ NA	Headache, hemiparesis left side	CT head: Right frontal hemorrhage CT venography: massive cerebral vein thrombosis with global edema and growth of hematoma	ICH, cerebral venous sinus thrombosis	Methylprednisolone, IVIG, thrombectomy, hemicraniectomy	Not improved, fatal

Mehta P.R. et. al./ UK Pt. 1	32 yr/ M	Astra-Zeneca	None, ex-smoker	9 days/ first dose	Thunderclap headache and subsequent left-sided incoordination and hemiparesis	CT head with venography: Superior sagittal sinus and cortical vein thrombosis and significant cortical oedema with small areas of parenchymal and subarachnoid hemorrhage	Cerebral Venous Sinus Thrombosis with SAH	None	Not improved, fatal
Mehta P.R. et. al./ UK Pt. 2	25 yr/ M	Astra-Zeneca	Primary sclerosing cholangitis, migraine, smoker	6 days/ first dose	Headache with photophobia, neck stiffness, and visual disturbances, associated with a non-blanching petechial rash over his lower limbs and bleeding of his gum, left hemiparesis and hemisensory loss, and focal motor seizures	CT head with venography: superior sagittal sinus thrombosis with extension into the cortical veins, and hemorrhage in lobar and subarachnoid locations	Cerebral Venous Sinus Thrombosis with SAH	Unfractionated Heparin, Dexamethasone, IVIG, Levetiracetam, platelet transfusions	Not improved, fatal
Castelli G.P. et. al./ Italy	50 yr/ M	Astra-Zeneca	None	11 days/ first dose	Severe headache, slight deviation of the right buccal rim, loss of strength in the right lower limb, unstable walking and slight visual impairment	CT head: intraparenchymal hemorrhage in the left hemisphere<br>CT angiography: multiple bleeding spots within the parenchymal hemorrhage and lack of opacification of the left transverse and sigmoid sinuses, suggesting thrombosis of the venous sinuses.	Cerebral Venous Sinus Thrombosis with ICH	Platelet transfusion, bilateral decompressive craniectomy	Not improved, fatal
Choi J.K. et. al./ Korea	33 yr/ M	Astra-Zeneca	None	9 days/ first dose	Headache, vomiting, tingling sensation in right arm, dysarthria, right hemiparesis.	CT head: subcortical hematoma in the left parietal lobe and an adjacent subarachnoid hemorrhage	Cerebral venous sinus thrombosis With ICH	Methylprednisolone, IVIG	Not improved, fatal

						MRI venogram: left sigmoid sinus and internal jugular vein were visualized, and the remaining dural venous sinus was not visualized, suggesting extensive CVT			
D'Agostino V. et. al./ Italy	54 yr/ F	Astra-Zeneca	NA	12 days/NA	Cerebrovascular accident-unspecified (DIC, MI, multi-district thromboses)	CT head: multiple intra-axial hemorrhages, with ipsilateral hemorrhagic subarachnoid suffusion, raising the suspicion of Labbè/superior longitudinal sinus thrombosis<br>MRI Brain: acute basilar thrombosis associated with the superior sagittal sinus thrombosis	Cerebral venous sinus thrombosis	None	Not improved, fatal
Jamme M. et. al./ France	69 yr/ F	Astra-Zeneca	HTN	11 days/ first dose	Headache with behavioral symptoms	CT head with MRI: severe bilateral frontal hemorrhage with brain herniation complicating a cerebral venous thrombosis of the left internal jugular vein, sigmoid sinus and superior sagittal sinus.	Cerebral venous sinus thrombosis	None	Not improved, fatal
Al-Mashdali A.F. et. al./ Qatar	32 yr/ M	Moderna	None	1 day/ first dose	Confusion, agitation, disorientation to time, place, person, and memory disturbances	MRI Brain: Normal<br>EEG showed findings suggestive of acute encephalopathy (slowed background activity).	Autoimmune encephalitis	Ceftriaxone and Acyclovir (failed), succeeded by IV Methylprednisolone	Improved
Liu, Benjamin D et. al./ USA<br>Pt. 1	86 yr/F	Moderna	CKD stage 3, Type 2 DM	7 days/ first dose	Acute confusion with visual hallucinations and left frontal headache	CT head and MRI brain: no acute findings<br>EEG: non-convulsive focal status seizure	Encephalopathy	Lorazepam and Fosphenytoin; Levetiracetam on discharge	Improved

Liu, Benjamin D et. al./ USA Pt. 2	73 yr/M	Moderna	Crohn's, hereditary hemochromatosis, HTN, and hyperlipidemia	7 days/ first dose	Unresponsiveness (staring episodes), restlessness, cognitive deficits, hallucinations, and confusion	CT head and MRI brain: no acute findings EEG: non-convulsive focal status seizure	Encephalopathy	Lorazepam and Levetiracetam	Improved
Chuang T.Y. et. al./ USA	45 yr/M	Moderna	None	5 days/ NA	Severe left-sided headache, unilateral eye pain with progressive ptosis, decreased vision, binocular diplopia, afferent pupillary defect and complete ophthalmoplegia	CT Head: Hyperattenuation of left cavernous sinus MRI brain and orbits and MRA/MRV head: Bilateral perineural enhancement surrounding optic nerve sheaths (left greater than right), plus single ill-defined enhancement in left orbital apex extending into the cavernous sinus	Tolosa-Hunt syndrome (THS)	Broad-spectrum antibiotics (discontinued), oral steroids	Improved
Torrealba-Acosta G. et. al./ USA	77 yr/ M	Moderna	CAD, hyperlipidemia, hypothyroidism	1 day/ first dose	Fever, generalized body rash, headache, dizziness, double vision, irregular orofacial movements, bilateral upper extremity myoclonus.	MRI Brain and Cervical spine: Normal CSF analysis: Cells: 120/mm3, protein: 124 mg/dl.	Acute encephalitis, myoclonus, sweet syndrome	Acyclovir, Methylprednisolone, antibiotics	Improved
Aladdin Y. et. al./ Saudi Arabia	42 yr/ F	Astra-Zeneca	NA	10 days/ first dose	Headache and subjective fever, generalized tonic-clonic seizure	MRI Brain: Subtle increase in the signal on FLAIR images at bilateral hippocampus and insula that was correlating with postictal changes	Refractory Status Epilepticus	Levetiracetam, Phenytoin, Lacosamide	Improved
Ghosh R. et. al./ India	68 yr/ M	Astra-Zeneca	HTN	4 days/ first dose	Impaired consciousness, bowel incontinence,	MRI Brain: Mild periventricular leukoaraiosis (left > right) and generalized cortical	Focal onset non-motor seizure	Brivaracetam	Improved

					behavioral disturbances	atrophy (predominantly affecting posterior cortex, right > left)			

M- male; F- female; NA- not available; TM- transverse myelitis; NMOSD- neuromyelitis optica spectrum disorder; LETM- longitudinally extensive transverse myelitis; ADEM- acute disseminated encephalomyelitis; RRMS- relapsing remitting multiple sclerosis; CVST- cerebral venous sinus thrombosis; SAH- subarachnoid hemorrhage; ICH- intracranial hemorrhage; DIC- disseminated intravascular coagulation; MI- myocardial infarction; THS- Tolosa-Hunt syndrome; DM- diabetes mellitus; CAD- coronary artery disease; CKD- chronic kidney disease; HTN- hypertension; MRC- medical research council; MRA- magnetic resonance arteriogram; MRV- magnetic resonance venogram; MRI- magnetic resonance imaging; FLAIR- fluid-attenuated inversion recovery; CT- computed tomography; EEG- electroencephalogram; CSF- cerebrospinal fluid; IVIG- intravenous immunoglobulin; PLEX- Plasmapheresis; HRT- hormone replacement therapy

Table S2. Studies with data from case reports and case series on COVID-19 vaccine and PNS associated manifestations.

**Author/ country**	**Patient age /gender**	**Type of COVID-19 vaccine**	**Comorbidities**	**Time Duration/ Doses to neurological symptom onset**	**Neurological presentation**	**Diagnostic Modality (RNS, single fiber EMG, NCS, MRI, muscle biopsy)**	**Diagnosis**	**Management**	**Outcome**
Waheed S. et. al./ USA	82/F	Pfizer	None	14 days/ second dose	Sustained fall due to weakness	Sagittal T1 post-contrast MRI lumbar spine demonstrating enhancement of cauda equina nerve roots	GBS	IVIG	Non-fatal/improved
Razok A. et. al./ Qatar	73/M	Pfizer	HTN, Rheumatoid arthritis	20 days/ second dose	Bilateral lower limb weakness	MRI of the spine showed bilateral nerve root enhancement in the lumbar region and the upper part of the cauda equina<br>CSF protein at 80 mg/dl and elevated albumin at 421 mg/L.<br>Nerve conduction study (NCS) and electromyogram (EMG) showed bilateral absent H reflexes in the	GBS	IVIG	Non-fatal/partially improved

						gastrocnemius muscles consistent with early polyneuroradiculopathy			
James J. et. al./ India Pt. 1	60/M	Astra-Zeneca	COPD	11 days/ first dose	Proximal weakness of the upper and lower limbs. Subsequently, numbness progressed and ascended up to the ankles in the lower limbs and up to the wrist in the upper limbs	Nerve conduction study (NCS) showed sensorimotor axonal neuropathy Cerebrospinal fluid (CSF) analysis showed elevated protein, normal sugar, and no cells	GBS	IVIG	Non-fatal/partially improved
James J. et. al./ India Pt. 2	66/M	Astra-Zeneca	NO	12 days/ first dose	Sensorimotor weakness Proximal lower limb with mild hand grip weakness	Nerve conduction study (NCS) showed demyelination with secondary axonopathy	GBS	IVIG, IVMP	Non-fatal/partially improved
James J. et. al./ India Pt. 3	54/F	Astra-Zeneca	Mastectomy for breast carcinoma	13 days/first dose	Sensorimotor weakness Proximal upper and lower limbs with weak hand grips. Trunk and neck weakness. Early respiratory involvement	Nerve conduction study (NCS) showed demyelination with secondary axonopathy	GBS	IVIG/IVMP	Non-fatal/partially Improved
Prasad A. et. al./ USA	41/M	Janssen	Morbid obesity	15 days	Facial diplegia	EMG showed a prolonged distal latency with conduction block and slow conduction velocity in bilateral tibial, peroneal nerve, and absent F waves. Csf showed albumino-cytological dissociation with	GBS (facial diplegia with paresthesia)	IVIG	Non-fatal/improved

						a CSF cell count of 50/uL (lymphocytes 91%, monocyte 9%, and CSF protein of 562 mg/dL. MRI lumbar spine showed thickening of cauda equina nerve roots			
Márquez Loza A.M. et. al./ USA	60/F	Janssen	None	10 days	Back and leg pain, diplopia, headache, bilateral facial weakness, numbness, areflexia	Brain MRI was normal. Lumbar puncture revealed the CSF had elevated protein (140 mg/dL), 9 nucleated cells, and glucose 63 mg/dL. Electrophysiologic studies showed normal compound muscle and sensory nerve action potentials and absent late responses (peroneal and posterior tibialis F-waves and H-reflexes) in the legs.	GBS	IVIG	Non-fatal/improved
Introna A. et. al./ Italy	62/M	Astra-Zeneca	HTN	2 days/first dose	Visual discomfort, ascending tetraparesis with proximal weakness, bilateral facial weakness, dysphagia, urinary retention, and distal paresthesia	Fundoscopy revealed severe bilateral optic disc edema CSF albumin-cytologic dissociation (total protein count 101 mg/dl, 5 white blood cells). Electrophysiologic study showed severe sensorimotor	GBS	IVIG	Non-fatal/ partially improved

						mixed polyneuropathy (demyelination with predominant axonal changes) Antiganglioside antibodies positive for IgG GM1 (titer 39)			
Suri V. et. al./ India	47/M	Astra-Zeneca	DM, HTN	17 days/first dose	Pure motor-flaccid quadriparesis with bilateral facial weakness with inability to stand without support	Brain MRI nonspecific hyperintensities in white matter in T2-FLAIR image. Spine MRI with contrast showed enhancing thickening of cauda equina nerve roots. NCV study, suggestive of acute inflammatory demyelinating polyradiculoneuropathy CSF revealed albuminocytological dissociation with protein of 250 mg/dL and glucose of 106 mg/dL and 0 cells.	Relapsing CIDP	IVIG, prednisolone and azathioprine	Non-fatal/ improved
Scedoni R. et. al./ Italy	82/F	Pfizer	AF/HTN	14 days/ second dose	progressive worsening of walking, with a lack of strength and sensitivity in the lower limbs.	Electroneurography results showed acute sensory-motor neuropathy of the demyelinating type. Electromyography showed decreased recruitment to the analysis of voluntary muscle activity, with signs of spontaneous	GBS (AMSAN)	IVIG	Non-fatal/ improved

						activity. Anti-ganglioside antibodies, in particular anti-sulfatide IgG (+) and IgM (++), anti-GM2 IgM (+) and anti-GM4 IgM (+) antibodies, were positive.			
Allen C.M. et. al./ UK Pt. 1	54/M	Astra-Zeneca	None	16 days/ first dose	Distal dysesthesia in his feet and hands, which ascended over two days, but had begun to recede as facial weakness developed	Electrophysiological assessment facial (NCS) showed severely reduced compound muscle action potential amplitude responses and normal terminal latencies bilaterally. The right orbicularis oris and oculi showed active denervation with no volitional motor activity. The left orbicularis oris and oculi showed active denervation with occasional fast firing, long duration polyphasic units and severely reduced recruitment. Sensory and motor NCS were normal in the upper and lower limbs CSF analysis revealed a mild lymphocytosis (19 cells / microlitre (ml), normal range ≤5) and elevated protein (162.6 mg/dl)).	GBS (facial diplegia with paresthesia)	Prednisolone	Non-fatal/ improved

						Normal MRI brain with contrast			
Allen C.M. et. al. /UK Pt. 2	20/M	Astra-Zeneca	Ulcerative colitis	26 days/ first dose	Developed dysesthesia in his distal lower limbs and three days before admission he developed facial diplegia	MRI of the brain was normal. CSF analysis revealed a mild lymphocytosis (14 cells / ml) and elevated protein (123.2 mg /dl). Facial NCS showed borderline normal amplitude responses and normal terminal latencies bilaterally. Bilaterally, orbicularis oculi and oris showed active denervation in addition to early recruited fast firing polyphasic units of small duration and low amplitude. Sensory and motor NCS were normal in the upper and lower limbs. Minimum F wave latencies were 28 ms in the right ulnar nerve and 49-50 ms in the tibial nerves.	GBS (facial diplegia with paresthesia)	Prednisolone	Non-fatal/ improved
Allen C.M. et. al. /UK Pt. 3	57/M	Astra-Zeneca	OA and Asthma	21 days/ first dose	Dull lumbar back pain radiating to flanks, followed by dysarthria, facial and proximal leg weakness, and distal dysaesthesia in feet	Sensory and motor NCS were normal in the upper and lower limbs. Non-contrast MRI of the brain was normal. CSF analysis revealed a mild	GBS (facial diplegia with paresthesia)	IVIG	Non-fatal/ improved

						lymphocytosis (8 cells / ml) and elevated protein (247.1 mg /dl).			
Allen C.M. et. al. /UK / Pt. 4	55/M	Astra-Zeneca	HTN	29 days/ first dose	Bilateral thigh paresthesias. One day later he reported numbness to the sacral and lumbar regions. Two days prior to admission he developed facial diplegia	MRI brain and whole spine with contrast showed enhancement of the facial nerve within the right internal auditory canal. CSF analysis revealed normal cell count (4 lymphocytes / ml) and elevated protein (89 mg / dl). NCS not performed	GBS (facial diplegia with paresthesia)	NA	Non-fatal/ improved
Burrows A. et. al./ UK	61/M	Pfizer	DM/ HTN/ dyslipidemia	1 day/first dose 2 days/ second dose	Right-sided lower motor neuron facial palsy with incomplete eye closure and no forehead movement	NA	Bell's palsy (sequential)	High dose steroid	Non-fatal/ improved
Repajic M. et. al./ USA	57/F	Pfizer	Bell's Palsy and HTN	2 days/second dose	Left-sided facial droop, left hyperacusis, ageusia, lagophthalmos, and facial soreness	NA	Bell's Palsy	Prednisone, antiviral agent	Non-fatal/improved
Colella G. et. al./ USA	37/M	Pfizer	None	5 days/ first dose	Left-sided facial droop, hemiparesis	Not done	Bell's Palsy	Prednisone, eye drops	Partial improvement
Yu B. et. al./ China	36/F	Sinovac	Bell's palsy	2 days/first dose	Right- sided facial weakness	CT head: normal	Bell's palsy	Prednisolone	Non-fatal/ improved
Ish S. et. al./ India	50/M	COVAXIN	None	21 days/ second dose	Right-sided facial palsy (LMN type)	NA	Bell's Palsy	Prednisolone	Non-fatal/ improved

Waqar W. et. al./ USA	57/F	Pfizer	None	7 days/ second dose	Intense burning dysesthesias in the feet, gradually spreading to the calves and minimally into the hands	An electrodiagnostic study performed on the day of presentation was normal. Skin biopsies showed multifocal involvement Hematoxylin and eosin and CD3 staining showed no histologic abnormalities.	Small fiber neuropathy	Anti-epileptic	Non-fatal/improved
Keir G. et. al./ USA	57/F	Pfizer	None	NA/second dose	Altered sense of smell -"Smelling smoke"- and headaches	axial T2 and FLAIR images there was T2 signal hyperintensity along the left olfactory bulb and bilateral olfactory tracts suggestive of edema	Phantosmia	NA	NA

M – Male; F – Female; GBS, Guillain-Barré syndrome; AMSAN - Acute motor and sensory axonal neuropathy; AMAN - Acute motor axonal neuropathy; BFP- Bifacial Diplegia; MFS - Miller-Fisher syndrome; NCS, Nerve conduction study; EMG, Electromyography; CSF, Cerebrospinal fluid; HTN, Hypertension; DM, Diabetes mellitus.

Table S3. Studies with grouped data with associated neurological manifestations with severity and outcomes for post COVID-19 vaccination.

Author/Country All Studies from	No. of Patients with Neuroimaging Findings	Vaccine type	Neurological Manifestation CNS = 1, PNS = 2	MRI Findings	Outcome (Non-Fatal = 1, Fatal = 2)
Hartmut Go ̈bel et.al/ Germany	2464	ChAdOx1 nCoV-19 (AZD1222) =2464	1= 2464 (Headache) 2=0	N/A	1=2464 2=0
Formeister EJ et.al/USA	40	Pfizer =28 Moderna =12	1=40 (Sensorineural hearing loss) 2=0	N/A	1=40 2=0
Pedro M Folegatti, et.al /UK	1077	ChAdOx1 nCoV-19 (AZD1222) =543 MenACWY=534	1=1077 (Headache) 2=0	N/A	1=1077 2=0
Krzywicka K et.al/ Netherland	213	ChAdOx1 nCoV-19 (AZD1222) =187 Pfizer =25 Moderna =1	1=213 (Cerebral venous sinus thrombosis) 2=0	N/A	1=167 2=46
Perry et.al /UK	95	ChAdOx1 nCoV-19 (AZD1222) =91 Pfizer =4	1=95 (Cerebral venous sinus thrombosis) 2=0	multiple venous infarction (10). multiple intracerebral hemorrhages (26) extracranial venous thrombosis (32)	1=41 2=54
Renoud Lucie et.al/France	844	Pfizer =749 Moderna =95	1=0 2=844 (Facial palsy)	N/A	1=844 2=0
Van Kammen et.al/ Amsterdam	116	ChAdOx1 nCoV-19 (AZD1222) =96 Pfizer=16 Sinovac=2 Moderna=1 Johnson & Johnson=1	1=116 (Cerebral venous sinus thrombosis) 2=0	intracerebral hemorrhage (58) thromboembolism (32)	1=58 2=58
Martina Patone, Lahiru et.al/UK	12,650	ChAdOx1 nCoV-19 (AZD1222) =1860 Pfizer= 1080 NA=9710	1=8779 (CNS demyelination, encephalitis, meningitis, myelitis, hemorrhagic stroke, SAH)	N/A	1=11,489 2=1161

	2=3871 (GBS, Bell's Palsy, Myasthenic disorder)	

CNS, Central nervous system; GBS, Guillain-Barré syndrome.
